# The Discovery of Novel Agents against *Staphylococcus aureus* by Targeting Sortase A: A Combination of Virtual Screening and Experimental Validation

**DOI:** 10.3390/ph17010058

**Published:** 2023-12-29

**Authors:** Kang Liu, Jiangbo Tong, Xu Liu, Dan Liang, Fangzhe Ren, Nan Jiang, Zhenyu Hao, Shixin Li, Qiang Wang

**Affiliations:** 1 College of Bioscience and Biotechnology, Yangzhou University, Yangzhou 225009, China; mz120211673@stu.yzu.edu.cn (K.L.); mx120221068@stu.yzu.edu.cn (J.T.); liangdanmolecule@163.com (D.L.); fzren@yzu.edu.cn (F.R.); 008036@yzu.edu.cn (N.J.); zhyuhao@hotmail.com (Z.H.); 2 College of Chemistry and Chemical Engineering, Yangzhou University, Yangzhou 225009, China; mx120210061@stu.yzu.edu.cn; 3 Department of the Heart and Great Vessels, Affiliated Hospital of Yangzhou University, Yangzhou 225009, China

**Keywords:** *Staphylococcus aureus*, Sortase A inhibitor, bacterial infection, drug screening

## Abstract

*Staphylococcus aureus (S. aureus)*, commonly known as “superbugs”, is a highly pathogenic bacterium that poses a serious threat to human health. There is an urgent need to replace traditional antibiotics with novel drugs to combat *S. aureus*. Sortase A (SrtA) is a crucial transpeptidase involved in the adhesion process of *S. aureus*. The reduction in virulence and prevention of *S. aureus* infections have made it a significant target for antimicrobial drugs. In this study, we combined virtual screening with experimental validation to identify potential drug candidates from a drug library. Three hits, referred to as Naldemedine, Telmisartan, and Azilsartan, were identified based on docking binding energy and the ratio of occupied functional sites of SrtA. The stability analysis manifests that Naldemedine and Telmisartan have a higher binding affinity to the hydrophobic pockets. Specifically, Telmisartan forms stable hydrogen bonds with SrtA, resulting in the highest binding energy. Our experiments prove that the efficiency of adhesion and invasion by *S. aureus* can be decreased without significantly affecting bacterial growth. Our work identifies Telmisartan as the most promising candidate for inhibiting SrtA, which can help combat *S. aureus* infection.

## 1. Introduction

*Staphylococcus aureus (S. aureus)* poses a significant threat to human health as it is a highly versatile and adaptable pathogen. The bacterium has the ability to produce a variety of virulence factors and toxins, such as hemolysins, enterotoxins, and superantigens, which contribute to its pathogenicity [1,2,3]. As we know, antibiotics are considered as effective drugs in combating microbes. However, their unrestrained use leads to an increase in bacterial pathogens’ resistance [4,5]. One of the most concerning aspects of *S. aureus* is its development of antibiotic resistance. Drug-resistant *S. aureus* strains have become increasingly prevalent, rendering it challenging to treat infections. These strains have developed resistance to multiple classes of antibiotics, making traditional treatment options less effective or even ineffective [6,7]. Conventional antibiotics always attack targets associated with cell death, which actually promote the development of drug resistance [8]. Over the past decade, researchers have studied another strategy for exploring drugs that specifically target the molecular determinants of virulence without bactericidal effects [9,10].

Adhesion to host tissue cells is the first and pivotal step in bacterial virulence, involving numerous cell wall proteins and protein complexes [11,12]. For *S. aureus*, the initial stage of the adhesion process involves the “recognition” between Sortase A (SrtA) and the LPXTG (Leucine-Proline-X-Threonine-Glycine, X represents any amino acid.) motif of MSCRAMMs (microbial surface components recognizing adhesive matrix molecules). Subsequently, SrtA catalyzes two cascade reactions, namely thioesterification and transpeptidation [13,14]. The correlation between SrtA and bacterial pathogenesis has been extensively explored. Studies on SrtA knockout mutants of *S. aureus* revealed a notable absence of renal abscesses and acute infections in mice [15]. Similar inhibitory effects on the virulence of *S. aureus* have been demonstrated in other studies [16,17,18]. Consequently, SrtA is considered a promising target for screening and designing novel antivirulence drugs aimed at interdicting bacterial adhesion [19,20].

Competitively occupying the functional sites is the primary approach in screening drugs for SrtA inhibition. The thiol catalytic residue of Cys184 serves as a target site, leading to the identification of certain drugs [21,22]. Another effective method involves competitively occupying more functional sites bound by the LPXTG motif. Various strategies have been devised to identify and characterize novel SrtA inhibitors [20]. High-throughput screening has gained widespread adoption for discovering new SrtA inhibitors, as it enables the identification of active molecules from thousands of compounds [23]. Additionally, computer-aided drug design (CADD) is also a cost-effective and less time-consuming method [24,25,26]. Particularly in recent years, advancements in computing power have facilitated the integration of multiple simulation methods for screening inhibitors from compound libraries.

In this work, we employed molecular docking, molecular dynamics (MD) simulation, MM/PBSA calculation, and experimental investigation to identify potential candidates from FDA (Food and Drug Administration)-approved drugs. Based on the docking binding energy and the presence of LPXTG motif-occupied sites, we identified three hits, namely, Naldemedine, Telmisartan, and Azilsartan. Stability analysis, including root mean square displacement (RMSD) and gyration radius, was conducted to assess the binding states during the dynamic process. Statistical analysis of hydrogen bonds and binding energy, along with energy decomposition based on the MD simulation trajectories, ultimately confirmed Telmisartan as the most promising candidate for SrtA inhibition. The antibacterial effect of Telmisartan was validated through assessments of bacterial growth, adhesion, and invasion. We also examined the toxicity of Telmisartan by exposing it to various cell types. Our findings suggest that Telmisartan shows promise as a SrtA inhibitor and has significant potential in combating *S. aureus* infections.

## 2. Results

### 2.1. Binding Pose of the LPXTG Sequence with SrtA

**Figure 1:**
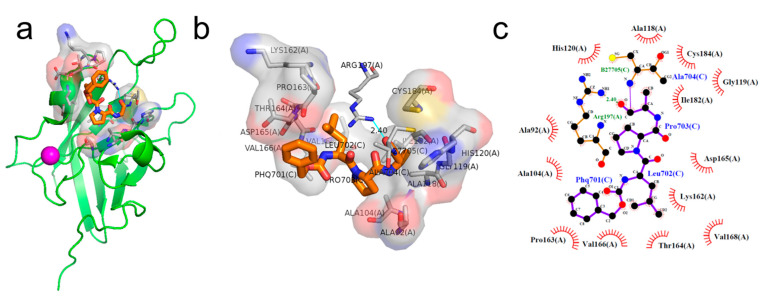
Analysis of the binding pose of the LPXTG sequence with SrtA (PDB ID: 2KID). (**a**) The structure of the complex. The binding pose of the LPXTG sequence with SrtA is depicted in 3D (**b**) and 2D (**c**) interaction diagrams. The light blue line indicates a hydrogen bond.

The inhibitors work by competitively binding to the functional sites of SrtA, specifically the sites that are bound by the LPXTG sequence. Analyzing the binding pose of the LPXTG sequence was our initial step in drug screening. The LPXTG–SrtA complex, a calcium-binding protein, is depicted in Figure 1a. The ligand is located within a hydrophobic cavity. The local binding mode was visualized using Pymol and is depicted in Figure 1b. The 2D interaction diagram, generated using LigPlot software (Version 2.2, EMBL, Hinxton, UK), depicts the binding pose of the LPXTG sequence with SrtA. It highlights the specific amino acids that are essential for establishing a stable binding state (Figure 1c). These functional amino acids include Ala92, Ala104, Ala118, Gly119, His120, Lys162, Pro163, Thr164, Asp165, Val166, Val168, Ile182, Cys184, and Arg197. Notably, Arg197 forms a hydrogen bond with the proline of the LPXTG fragment. The active sites of the Sortase family contain three conserved residues: His120, Cys184, and Arg197. The catalytic importance of these residues has been extensively proven [27,28,29,30]. Previous studies have also confirmed the importance of other residues, including Val168, Ala104, and Ala118 [31]. The interaction sites between SrtA and LPXTG serve as valuable references for our subsequent drug screening efforts.

### 2.2. Virtual Screening using Molecular Docking

A total of 2115 FDA-approved drugs were selected for molecular docking with SrtA. The chemical compounds were ranked based on their binding energy, with a lower docking binding energy indicating a higher affinity. A value of −9.0 kcal/mol was selected as the threshold value. In the FDA-approved drug library, 12 drugs were initially selected as candidates due to their binding energies being lower than −9.0 kcal/mol. The maximum binding energy was −9.6 kcal/mol (Table 1). Interestingly, the majority of the screened drugs exhibit similar structural scaffolds, including benzene rings and benzodiazepine rings.

While the docking results are not directly related to drug effect, a higher docking score, such as its binding energy, generally suggests a greater probability of potentially inhibiting the enzyme’s activity. Apart from the docking score, the binding region is crucial for the efficacy of an inhibitor. A more promising inhibitor should bind to the active cavity and block more functional sites [32]. Therefore, we analyzed the binding poses of the selected drugs using the LigPlot software. A summary of all binding sites can be found in Table S1, while the 2D and 3D binding modes are illustrated in Figures S1–S12. Taking the example of the top drug, with ID number 1, although it has the highest binding energy, it does not target any functional sites of the SrtA, making it unsuitable as a SrtA inhibitor. We further analyzed the ratio of occupied sites, considering the marginal differences in the binding energies among these compounds. Only three drugs had an occupation rate exceeding 70%: Naldemedine, Telmisartan, and Azilsartan (Table 1 and Table S1). As discussed earlier, Cys184 emerged as a pivotal functional site. Should the drugs establish stable binding to this site, they would demonstrate the potential to impede the thioesterification reaction. Therefore, we also paid attention to Triazolam, Midazolam, and Alprazolam, which could bind to Cys184 as indicated by the molecular docking. However, the stability of the binding to Cys184 requires further scrutiny. The MD results reveal that these drugs fail to bind consistently to Cys184 throughout the 100 ns simulations, suggesting weak inhibitory potential (Figure S15). Consequently, our subsequent analysis focused solely on the three drugs that occupy more functional sites.

**Figure 2:**
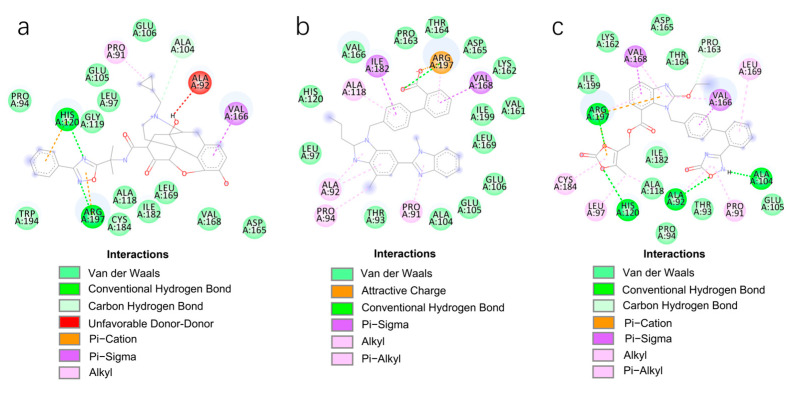
Two-dimensional interaction diagrams of the binding poses of three candidate drugs with SrtA. (**a**) Naldemedine, (**b**) Telmisartan, and (**c**) Azilsartan.

An extensive interaction analysis was conducted using Discovery Studio to identify the binding modes of the three screened drugs with SrtA. The binding poses were visualized using 2D interaction diagrams, as shown in Figure 2. The main forces that drive these binding poses are hydrophobic interactions and hydrogen bonds. Most amino acids interact with Naldemedine through van der Waals interactions. Specifically, His120 and Arg197 can form conventional hydrogen bonds with nitrogen atoms. Additionally, these two amino acids can also form two pi–cation interactions. Moreover, Ala104 can form a carbon–hydrogen bond with a hydroxy group. The binding energy is also influenced by pi–sigma and alkyl interactions involving Val166 and Pro91, respectively. Unfavorable interactions between donors can negatively impact the stability of binding during the dynamic process (Figure 2a). Telmisartan can form more pi–alkyl, alkyl, and pi–sigma interactions compared to Naldemedine. The van der Waals interaction is the primary force for most amino acids. Notably, Arg197 can form both charge attraction and conventional hydrogen bonds with the same carboxyl group. As we know, charge attraction is a powerful interaction; therefore, we speculate that this robust binding could impede the formation of hydrogen bonds between Arg197 and the proline of the LPXTG fragment (Figure 2b). For Azilsartan, while there are fewer van der Waals interactions, there is an increase in pi–alkyl and alkyl interactions. Ala92, Ala104, His120, and Arg197 can form H-bonds with oxygen atoms or amidogen. Additionally, Arg197 can participate in two pi–cation interactions, while pi–sigma interactions are facilitated by Val166 and Val168 (Figure 2c).

### 2.3. Stability Analysis of the Binding Poses

**Figure 3:**
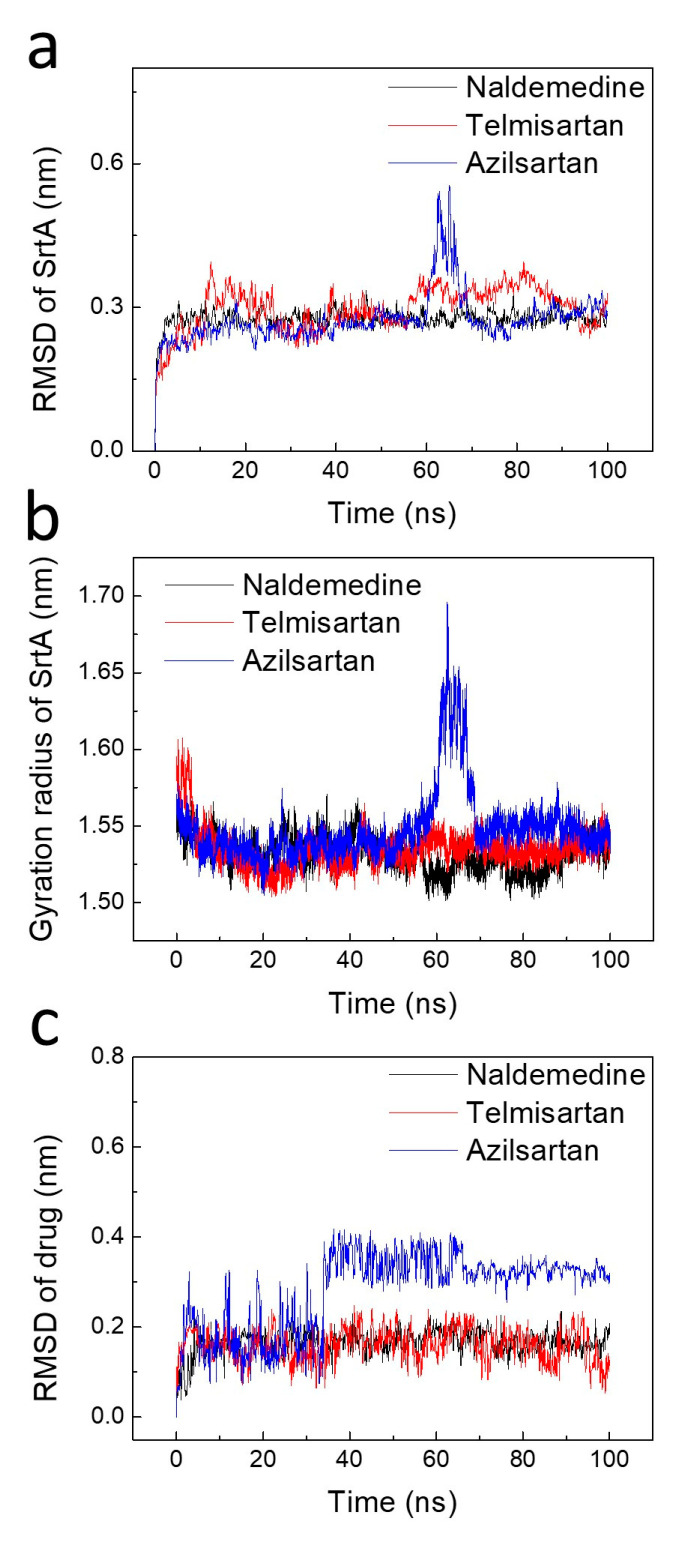
The stability analysis of the binding poses of the three candidate drugs indicates that the binding of Telmisartan is most stable. The time sequence of the RMSD (**a**) and gyration radius (**b**) of SrtA. (**c**) Time evolutions of the RMSD of the drugs.

Molecular docking is a static simulation that aims to find the optimal conformation using a scoring function or the binding energy [33]. However, in reality, the binding of inhibitors to a reporter is a dynamic process. Therefore, these binding poses require stability assessment using molecular dynamics simulations. The RMSD of SrtA initially increases continuously for all three compounds, eventually reaching an equilibrium at around 0.3 nm. The RMSD of SrtA for Azilsartan shows a sudden fluctuation after 60 ns but stabilizes within the next 10 ns (Figure 3a). In Figure 3b, the gyration radius of SrtA is depicted. Throughout the 100 ns MD simulations, all complexes display a reduction, indicating stronger binding between the protein and drug. A sudden fluctuation of the gyration radius is also observed in the Azilsartan system, which corresponds to the RMSD of SrtA. Based on the higher RMSD of Azilsartan (Figure 3c), Naldemedine and Telmisartan are more stable.

### 2.4. Binding Strength Assessment

**Figure 4:**
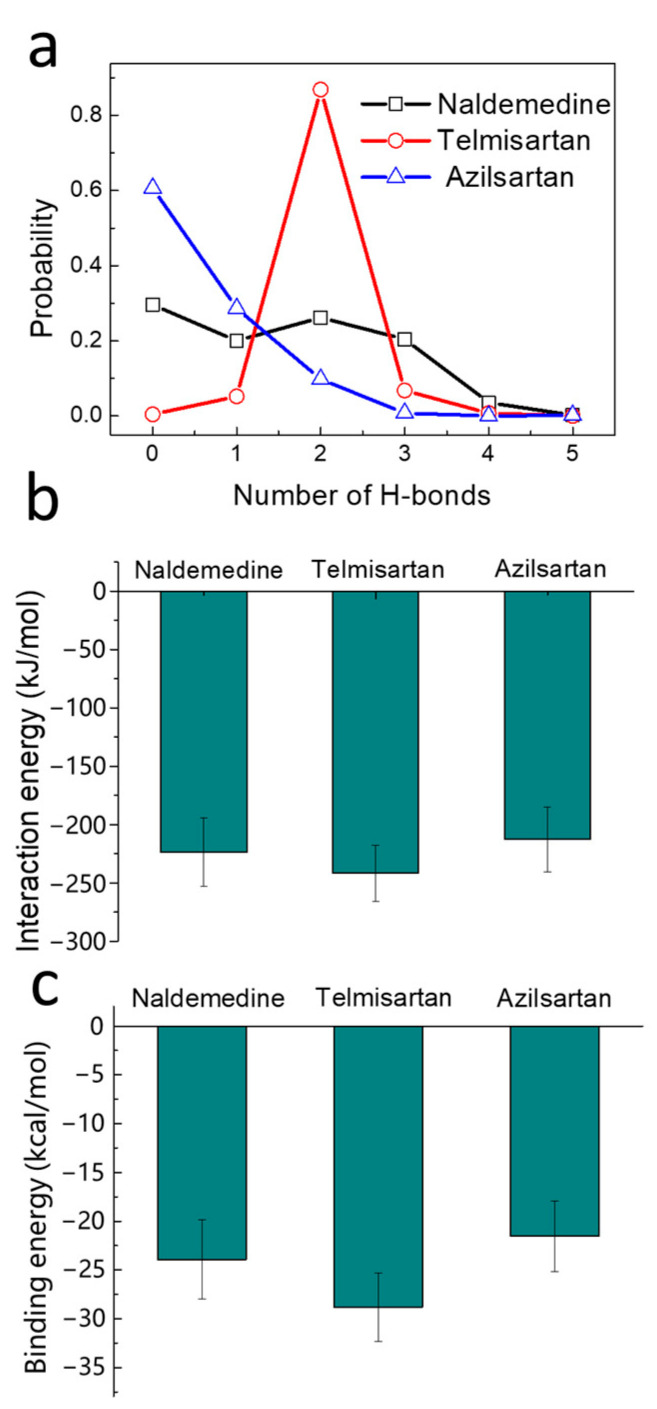
Assessment of the drug binding strength demonstrates a higher affinity for binding in the case of Telmisartan. Probability statistics of H-bond number (**a**) and interaction energy (**b**) based on the last 50 ns of the MD simulations. (**c**) Binding energy calculated using the MM/PBSA method.

In addition to conformation analysis, we calculated the binding strength based on the MD trajectories. The probability statistics of hydrogen bond numbers indicate that Telmisartan can consistently form two stable hydrogen bonds, while Naldemedine and Azilsartan struggle to form stable hydrogen bonds. The probability of Naldemedine having zero H-bonds is as high as 60%, indicating the weak stability of the binding state (Figure 4a). The interaction energy, comprising coulombic potential and LJ potential, was calculated using Gromacs software (Version 2019.3). The average was taken from the final 20 ns of the simulation. The interaction between Telmisartan and SrtA is the strongest among the compounds, as shown in Figure 4b, indicating an optimal inhibitory effect. The binding energy was then calculated using the MM/PBSA method, taking the solvent effect into account. The advantage of Telmisartan still exists, with a binding energy of −28.8 kcal/mol (Figure 4c).

**Figure 5:**
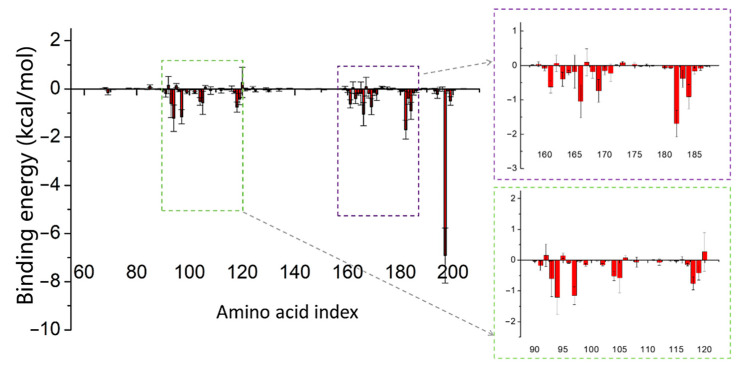
The energy decomposition of all amino acids of SrtA bound with Telmisartan.

MM/PBSA calculations were conducted to investigate the dynamic binding states and determine the contributions of each amino acid. For Telmisartan (Figure 5), notable contributions to the binding energy for Thr93, Pro94, Leu97, Ala104, Glu105, Ala118, Val161, Val166, Leu169, Cys184, and Arg197 were observed, all of which were lower than −0.5 kcal/mol. Arg197 even exhibited a significant binding energy contribution of −6.7 kcal/mol, which is supported by our previous findings (Figure 2b). There were no apparent repulsive interactions contributed by any amino acids, and the binding energy of most amino acids was negative. Distinct repulsive interactions by amino acids were observed for Naldemedine and Azilsartan (Figures S14 and S15). These repulsive interactions act as destabilizing factors, potentially weakening the inhibitory effect.

### 2.5. Efficacy Evaluation of Telmisartan on Bacterial Growth and Infection

**Figure 6:**
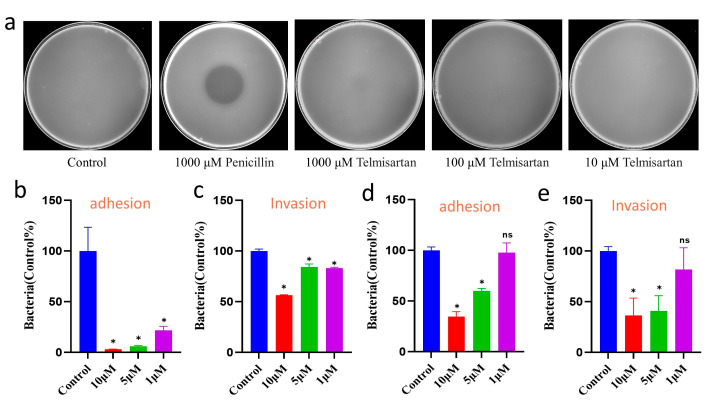
Efficacy evaluation of Telmisartan on the growth and infection of *S. aureus* reveals that Telmisartan is capable of reducing infection without impacting bacterial growth. (**a**) The antibacterial effect test of Telmisartan with various concentrations (1000 μM, 100 μM, and 10 μM) and 1000 μM penicillin against the RN4220 strain. The sample incubated with only DMSO was used as a control. Adhesion and invasion assay of RN4220 (**b**,**c**) and USA300 strains (**d**,**e**). The data (**b**–**e**) presented in this study represent the mean ± standard error of the mean (SEM) obtained from three independent experiments. Statistical significance was assessed by employing a two-tailed Student’s *t*-test. * *p* < 0.05.

Utilizing simulation techniques, we have identified a promising candidate called Telmisartan. To evaluate the antibacterial efficacy of Telmisartan on the growth of *S. aureus* (RN4220 strain), we measured the size of the inhibition zone. As shown in Figure 6a, a distinct inhibition zone was observed when *S. aureus* was exposed to 1000 μM penicillin. However, Telmisartan exhibited no significant inhibitory effects on *S. aureus*. The MIC (minimum inhibitory concentration) reached 1000 μM (Figure S18 and Table S2). Given the significant role of SrtA in bacterial adhesion [13,14], we further examined the adhesion and invasion of *S. aureus* in HEK293 cells. In Figure 6b, it is shown that even a lower concentration of 1 μM Telmisartan substantially inhibited adhesion efficiency for the RN4220 strain. Consequently, the ability of *S. aureus* to invade cells was weakened, particularly at higher concentrations (Figure 6c). Bacterial drug resistance has become a growing concern in recent years, with various drug-resistant bacteria emerging [6,7]. We questioned whether Telmisartan, identified in our screening, would be effective against drug-resistant bacteria. To address this, we selected a penicillin-resistant strain (USA300 strain) to evaluate the effects of Telmisartan on adhesion and invasion. To our surprise, Telmisartan at concentrations of 1000 μM, 100 μM, and 10 μM not only inhibited the adhesion process but also decreased cell invasion (Figure 6d,e). Additionally, the effect on biofilm formation was also tested. The Abs_570_ value initially remained relatively stable and then increased as concentrations increased up to 125 μM, which subsequently decreased sharply at concentrations of 250 μM and further reduced at concentrations of 500 μM. This suggests that Telmisartan also interferes with the biofilm formation of *S. aureus* (Figure S20).

### 2.6. Toxicity Assay of Telmisartan to Various Cell Types

**Figure 7:**
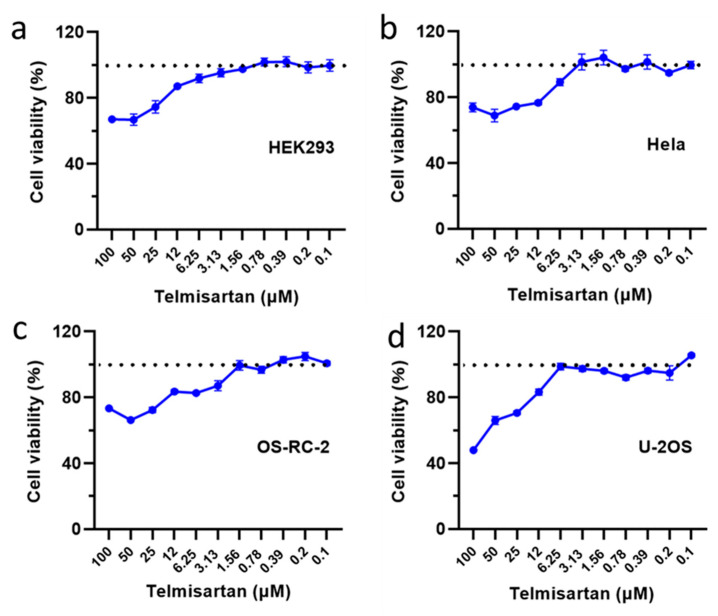
Toxicity assay suggests that Telmisartan has minimal toxicity at recommended clinical dosages. (**a**) HEK293 cell, (**b**) Hela, (**c**) OS-RC-2, and (**d**) U-2OS. The bar graphs display the mean values from three separate experiments, along with their corresponding standard deviations (SD).

We not only studied the effects of Telmisartan on bacterial growth and infection, but also conducted a thorough evaluation of its toxicity on different types of cells. Among our tests, all cells remained viable when exposed to concentrations below 1 μM, as shown in Figure 7. Telmisartan significantly inhibited the adhesion process of *S. aureus* to HEK293 cells at a concentration of 1 μM (Figure 6b,d). Even at a concentration of 5 μM, the majority of cells remained active (Figure 7). At this concentration, both bacterial adhesion and invasion were significantly reduced, especially for the penicillin-resistant strain USA300 (Figure 6e). As the concentration increased further, cell viability gradually decreased. However, the rate of decline was relatively slow, and some cells eventually reached a plateau (Figure 7a–c). This suggests that Telmisartan has minimal toxicity at recommended clinical dosages.

## 3. Discussion

The virulence factor SrtA is crucial in the pathogenesis of Gram-positive superbugs [19,20]. Over recent years, researchers have investigated various SrtA inhibitors to counteract infections caused by Gram-positive superbugs, such as *S. aureus*. These inhibitors encompass synthetic small molecules, peptides, and various natural products [34]. Within the realm of small molecule inhibitors, two fundamental molecular mechanisms have been revealed. Pyridazinone-based molecules have been demonstrated to covalently modify the active Cys thiol of SrtA, directly disrupting its function [35]. Furthermore, specific inhibitors mimic the natural SrtA substrate, causing conformational changes around the active sites [36]. Structure-based approaches have emerged as a strategic choice for studying small molecules as inhibitors of SrtA biological activity.

Exploring new drugs requires substantial financial investment and time commitment. In this study, we integrated virtual screening and experimental validation to identify potential drug candidates from a drug library based on their active sites. Utilizing an FDA-approved drug library offers a cost-saving advantage [37,38], but the limited number of drugs may decrease the likelihood of successfully targeting SrtA. Fortunately, our efforts led to the identification of Telmisartan as a promising drug candidate. Even at low, non-toxic concentrations, Telmisartan significantly reduces the adhesion and invasion of *S. aureus*. The noteworthy aspect of our findings is that Telmisartan, at micromolar levels, significantly inhibited the adhesion efficiency of *S. aureus*. This dosage is notably lower than that required by other previously studied inhibitors, such as chlorogenic acid [39] and aryl 3-acryloamide [36]. It is worth noting that the mean ± SD value for Cmax was 159 ± 104 ng/mL (or 0.3 ± 0.2 µmol/L) for Telmisartan at 40 mg when used for hypertension [40]. This dosage is indeed too low relative to the content used in antibacterial tests. As an alternative approach, combining antivirulence drugs with traditional antibiotics has proven to be a more effective strategy in bacterial defense [41]. Therefore, we conducted new investigations into the combined use of antibiotics and Telmisartan against *S. aureus*. The results indicate that combined administration can reduce the dosage of penicillin needed to inhibit the growth of *S. aureus* (Figure S19). At the clinical dosage of 0.3 μM of Telmisartan, the MIC can also be reduced from 1.95 μM to 0.98 μM, and the FICI (fractional inhibitory concentration index) can reach 0.5, suggesting a typical synergy effect (Table S2).

In fact, Telmisartan has been reported earlier for its defensive capabilities against various bacteria, including Gram-negative [42] and Gram-positive bacteria [41]. For Escherichia coli K1, Telmisartan has been shown to inhibit their invasion into human brain microvascular endothelial cells by targeting the angiotensin II receptor type 1 [42]. The mechanism is different from our work that focused on a crucial target called SrtA, a critical enzyme in Gram-positive bacteria. Interestingly, a previous paper also reports that Telmisartan can potentially target the NorA efflux pump, resulting in an increased intracellular concentration of antibiotics, thereby restoring their antibacterial activity against *S. aureus* and cell susceptibility [41]. It appears that our work provides a new mechanism for inhibiting *S. aureus*, suggesting that Telmisartan can target both the NorA efflux pump and SrtA to defend against the *S. aureus* infections.

Another point that needs to be discussed is the method used in our work. Although we did not demonstrate, in a biological setting, the inhibition of SrtA using the compounds, molecular docking, MD simulation, and MM/PBSA calculation were employed to investigate the interactions between these compounds and SrtA. These methods have been identified to accurately calculate the binding strength between drugs and proteins [43,44]. However, it is known that these simulation systems are relatively simplistic, presenting a certain deviation from reality. Consequently, we conducted experimental investigations to determine whether these compounds can inhibit adhesion and invasion. Combing theoretical prediction with pharmacodynamic experiments is a recently developed and effective method to screen and design novel drugs [45,46].

While Telmisartan has been identified as a promising candidate for SrtA inhibition, it is important to note that its molecular structure may not be optimal. Our next step is to use the results from the binding pose insights and incorporate knowledge from other inhibitors. By combining and modifying the structural elements of Telmisartan with novel functional units, we aim to improve its effectiveness as a SrtA inhibitor. Integrating diverse structural elements and adding new functional groups could potentially improve the binding affinity and specificity against SrtA. This iterative process of structural optimization aligns with the principles of rational drug design, involving successive modifications to enhance the therapeutic properties of a lead compound. We expect to acquire a stronger and more selective SrtA inhibitor, furthering our pursuit of effective strategies against *S. aureus*.

## 4. Materials and Methods

### 4.1. Structural Preparation for Simulations

The initial structures of SrtA were obtained from the Protein Data Bank (https://www.rcsb.org, accessed on 28 December 2022). The PDB ID for this structure is 2KID. The structure of the Bank does not include an integrated SrtA, as it is missing the embedded part (AA 1-58) within the membrane. Thus, we restricted the AA 59 of SrtA to simulate the membrane-bound state in the molecular dynamics simulations. The molecular structures of the FDA-approved drug library, comprising 2115 compounds, were obtained from the ZINC database (https://zinc.docking.org/, accessed on 28 December 2022). Then, the ligands were prepared using LigPrep with the default settings at pH 7.2 ± 1.0.

### 4.2. Molecular Docking Simulation

One enzyme (SrtA) and 2115 ligands were prepared using AutoDockTools [47] to serve as the input files for the docking simulation. Additionally, two inhibitors were selected as positive (IC50 7.24 μg/mL) [48] and negative (IC50 > 1000 μM) [49] controls, respectively. For the receptor protein, all water molecules, ligands, and ions were removed, and polar hydrogens were added. The Kollman United charge method was used to calculate the partial atomic charge. The OpenBabel [50] software (Version 3.1.1) was used to separate the files, add hydrogen bonds and assign rotatable bonds and Gasteiger–Marsili charges, and finally save these ligands in PDBQT format.

All molecular docking simulations were performed using AutoDock Vina [51] (version 1.1.2). Based on the crystal structure, the ligand search space for docking encompassed the entire protein surface. The grid box was set as 49.3 × 45.6 × 38.3 nm^3^. Ten binding conformations were provided for each complex based on the docking binding energy. The binding energy indicates the affinity between the receptor and the ligand, which is an important indicator for drug screening. After conducting molecular docking simulations, the binding poses were analyzed using LigPlot [52] and Discovery Studio [53]. The screened complexes were analyzed using binding energy and binding pose analysis, and then subjected to molecular dynamics simulations for further assessment and analysis. The binding energy was corrected for based on molecular weight (MW), which was represented with the corrScore [54].

### 4.3. Molecular Dynamics Simulation

All simulations were conducted using the GROMACS 2019-3 software package [55]. The drug StrA complex, along with Ca^2+^, was initially positioned at the center of a cubic box with a distance of 1.5 nm between the complex and boundary. The complexes were solvated in TIP3P water and neutralized by adding NaCl. The Amber force field was chosen for our simulation. The force field of drug molecules was generated using CGenFF tools [56]. The system was energy-minimized using the steepest descent method until convergence was reached. After reaching the energy minimum and achieving quick equilibrium in the NVT ensemble (constant bead number, volume, and temperature) for 1 ns, the systems were then carried out under the NPT ensemble, with a constant bead number, pressure, and temperature. The pressure was maintained at a constant 1 bar using the isotropic Parrinello–Rahman barostat, and the temperature was kept at 310 K using the Nose–Hoover thermostat, with a coupling constant of 4 ps. A cutoff of 1.2 nm was used to define short-range interactions. The Lennard–Jones potential was smoothly shifted to zero between 0.9 nm and 1.2 nm to minimize the cutoff noise. The particle-mesh Ewald summation method was used to handle long-range electrostatic interactions. Periodic boundary conditions were applied in all three directions. The time step was 10 fs, and the neighbor list was updated every ten steps. All visualizations were created using PyMOL [57].

### 4.4. MM/PBSA Method

The binding energy between StrA and the drug molecules was calculated by the following formula:

$$\Delta E_{\mathrm{binding}}=\Delta E_{ele}+\Delta E_{vdw}+\Delta E_{PB}+\Delta E_{SA}$$

where $\Delta E_{ele}$ , $\Delta E_{vdw}$ , $\Delta E_{PB}$ , and $\Delta E_{SA}$ are the electrostatic energy, van der Waals energy, polar term of solvation, and nonpolar term of solvation, respectively. Two hundred frames of the last 20 ns of the simulation trajectories were used to calculate the binding energy in each system using the screening molecular mechanics/Poisson–Boltzmann surface area (MM/PBSA) method [58,59,60]. All the MM/PBSA calculations were performed using the gmx_mmpbsa script (https://github.com/Jerkwin/gmxtools/, accessed on 20 January 2023). This script utilized the APBS software (Version 3.4.0) (https://github.com/Electrostatics/apbs/, accessed on 20 January 2023) to solve the Poisson–Boltzmann equation and calculate the polar term and nonpolar term of solvation energy.

### 4.5. Antibacterial Efficiency Assay

*S. aureus* (RN4220 strain, obtained from Professor Jinlin Huang, Yangzhou University) was initially inoculated into a liquid culture medium and incubated at 37 °C until it reached the logarithmic growth phase (OD_600_ = 1.0). A specific volume of the bacterial suspension was injected into pre-cooled agar medium at approximately 50 °C, mixed thoroughly, and poured onto Petri dishes (approximately 20 mL per dish). We then let the agar solidify, and then added 10 µL of the test reagent (1000 μM, 100 μM, and 10 μM) to the center of each plate. The reagent was then allowed to dry in a horizontal position. The plates were then incubated for 24 h at 37 °C, and the inhibition zones were observed using BIO-RAD GelDoc Go (BIO-RAD, Hercules, CA, USA).

### 4.6. MIC and Biofilm Formation Tests

This experiment followed the microdilution protocol according to CLSI (Clinical and Laboratory Standards Institute, 2015), using Tryptic Soy Broth (TSB) and a U-shaped 96-well plate. Telmisartan was prepared in the 96-well plate in 19 double-fold serial dilutions, with the concentration ranging from 4000 μM to 0.03 μM. *S. aureus* (RN4220 strain) was grown overnight on Tryptone Soy Agar (TSA) plates and then suspended in TSB. The optical density (OD) was adjusted to 0.5. The diluted bacteria were then added to the wells containing Telmisartan, resulting in a final concentration of 5 × 10^5^ CFU/mL. After overnight incubation at 37 °C, the MIC was determined with visual inspection [61]. Then, the 96-well plate was washed twice with sterile PBS, inverted to air-dry, and stained with 1% crystal violet for 15 min. Subsequently, wells were rinsed with sterile PBS, treated with elution solution (30% acetic acid) for 15 min, and the absorbance at 570 nm was measured using a Spark microplate reader (TECAN, Zurich, Switzerland) [62].

### 4.7. Bacterial Adhesion and Invasion Assay

First, HEK293 cells (ATCC, Manassas, VA, USA) were seeded in 24-well plates with Dulbecco’s Modified Eagle Medium (DMEM) containing 10% Fetal Bovine Serum (FBS) at a cell density of 2 × 10^5^ cells per well. The cells were then incubated at 37 °C with 5% CO_2_ for 12 h. RN4220 and USA300 strains (obtained from the laboratory of Professor Jinlin Huang, Yangzhou University) were inoculated into liquid culture medium and grown at 37 °C until they reached the logarithmic growth phase. The bacterial cultures were collected via centrifugation at 5000 rpm and 4 °C for 5 min. They were then washed three times with sterile phosphate-buffered saline (PBS), and the bacterial pellets were then collected. The bacteria were enumerated and used to infect various cells at a multiplicity of infection (MOI) of 100:1. Telmisartan (MCE, Junction, NJ, USA) was added to the bacterial suspension at the desired concentration, and dimethyl sulfoxide (DMSO) was added as a control group (with at least three parallel samples). The HEK293 cells were washed three times with DMEM without FBS and penicillin streptomycin solution (P/S). Then, the diluted bacterial suspension was added to the wells containing the cultured cells. The cells were incubated at 37 °C in a 5% CO_2_ incubator for 2 h. The cells were washed three times with sterile PBS to remove non-adherent bacteria. For the adhesion assay, 100 µL of 0.5% Triton X-100 was added to lyse the cells. For the invasion assay, DMEM containing antibiotics (150 μg/mL) was added for 2 h to eliminate extracellular bacteria before lysing the cells. The cell lysate was diluted, plated on solid culture medium, and used for bacterial counting [63,64].

### 4.8. Combined Administration Tests

Using Tryptic Soy Broth (TSB) and a U-shaped 96-well plate, penicillin was prepared in 12 two-fold serial dilutions, resulting in a final concentration range of 125 μM to 0.06 μM. The highest concentration was in the first well of each row, and the lowest concentration was in the twelfth well. The bacterial suspension was diluted as in the MIC experiment mentioned above. Telmisartan was separately added to the diluted bacteria at final concentrations of 0.3, 10, or 100 μM. The synergistic MIC (the first well without visible turbidity) was determined with visual inspection. Absorbance at 600 nm was measured using a Spark microplate reader. The observed MIC values were used to calculate the FICI, based on the following formula: $FICI=\frac{MIC\;of\;antibiotic\;and\;compound}{MIC\;of\;antibiotic\;alone}+\frac{MIC\;of\;antibiotic\;and\;compound}{MIC\;of\;compound\;alone}$ [65].

### 4.9. Drug Toxicity Assay

Cells were seeded in a 96-well plate at a density of 1 × 10^4^ cells per well, with 100 μL in each well. After 24 h of incubation, Telmisartan was dissolved in 1% DMSO to achieve final concentrations ranging from 100 μM to 50, 25, 12.50, 6.25, 3.13, 1.56, 0.78, 0.39, 0.20, and 0.10 μM. The solution was added to the cells for an additional 24 h. The cell viability of the treatment groups was assessed at different concentrations using a CCK-8 assay kit (MCE, Junction, NJ, USA). The formula for calculating cell viability is as follows: Viability = (Drug treatment group/Control group) × 100%.

### 4.10. Statistical Analysis

The statistical analyses were conducted using IBM SPSS Statistics software (Version IBM SPSS 27, IBM SPSS, Armonk, IL, USA). Statistical differences were assessed using one-way ANOVA with Dunnett’s post hoc tests or unpaired two-tailed Student’s t-tests for multiple comparisons. *p*-values less than 0.05 were used to indicate statistical significance and are displayed in the figures. Non-statistically significant differences are abbreviated as “ns”.

## 5. Conclusions

SrtA is a crucial target for antivirulence strategies against *S. aureus*. Utilizing existing drug repositories to screen for SrtA inhibitors is an efficient strategy. In this study, we combine theoretical calculations and experimental investigations to identify the potential candidates from FDA-approved drugs. Three promising candidates, Naldemedine, Telmisartan, and Azilsartan, were identified based on their docking binding energy and the ratio of occupied SrtA sites. The primary forces driving the binding were hydrophobic interactions, hydrogen bonds, and charge attraction. MD simulations and MM/PBSA calculations demonstrated that Telmisartan has a strong and stable binding affinity to SrtA. Telmisartan was shown to reduce the adhesion and invasion of *S. aureus* at 10 μM without significant cytotoxicity. Our research identifies Telmisartan as the most promising candidate for inhibiting SrtA and it has significant potential in combating bacterial infections caused by *S. aureus*.

## Figures and Tables

**Table 1:** The molecular docking results indicate that the top 12 drugs have binding energies below −9.0 kcal/mol. The positive and negative controls are depicted in the final two lines. The occupied sites correspond to the binding sites of LPXTG, as shown in Figure 1c.

ID	Compound	Structure	Binding Energy (kcal/mol)	corrScore	Ratio of Occupied Sites
1	Trypan Blue	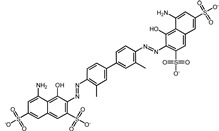	−9.6	0	0
2	Naldemedine	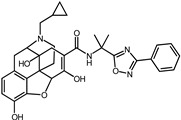	−9.6	0.84	71.4%
3	Lomitapide	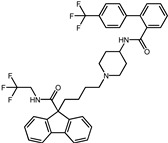	−9.4	0.69	64.3%
4	Norgestrel	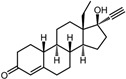	−9.2	0.86	0
5	Triazolam	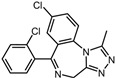	−9.1	0.82	50.0%
6	Flourescein	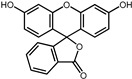	−9.1	0.82	57.1%
7	Midazolam	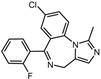	−9.0	0.80	64.3%
8	Simeprevir	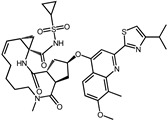	−9.0	0.55	50.0%
9	Alprazolam	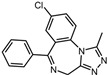	−9.0	0.80	50.0%
10	Telmisartan	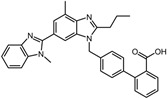	−9.0	0.72	71.4%
11	Nilotinib	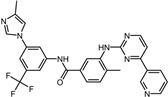	−9.0	0.71	64.3%
12	Azilsartan	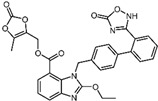	−9.0	0.69	85.7%
Positive control	Rosmarinic acid	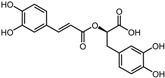	−7.6	0.39	64.3%
Negative control	2,3-Bis(4-methoxyphenyl)propanenitrile	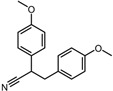	−6.2	0.18	35.7%

## Data Availability

All data are contained within the article.

## Supplementary Materials

The following supporting information can be downloaded at: www.mdpi.com/xxx/s1, Figures S1–S12: Analysis of the binding poses of the top 12 drugs with SrtA. Figures S13 and S14: Analysis of the binding poses of two controls with SrtA. Figure S15: The most stable binding poses of Triazolam, Midazolam, and Alprazolam with SrtA. Figure S16: The energy decomposition of all amino acids of SrtA bound with Naldemedine. Figure S17: The energy decomposition of all amino acids of SrtA bound with Azilsartan. Figure S18: The effect of serial concentrations of Telmisartan on the growth of *S. aureus*. Figure S19: The effect of combination administration on the growth of *S. aureus*. Figure S20: The effect of serial concentrations of Telmisartan on the biofilm formation of *S. aureus*. Table S1: Binding sites of 12 screened drugs and two controls. Table S2: The results of the minimum inhibitory concentration (MIC) and fractional inhibitory concentration index (FICI).
