# Correction: Liu et al. The Discovery of Novel Agents against *Staphylococcus aureus* by Targeting Sortase A: A Combination of Virtual Screening and Experimental Validation. *Pharmaceuticals* 2024, *17*, 58

**DOI:** 10.3390/ph17040453

**Published:** 2024-04-01

**Authors:** Kang Liu, Jiangbo Tong, Xu Liu, Dan Liang, Fangzhe Ren, Nan Jiang, Zhenyu Hao, Shixin Li, Qiang Wang

**Affiliations:** 1 College of Bioscience and Biotechnology, Yangzhou University, Yangzhou 225009, China; mz120211673@stu.yzu.edu.cn (K.L.); mx120221068@stu.yzu.edu.cn (J.T.); liangdanmolecule@163.com (D.L.); fzren@yzu.edu.cn (F.R.); 008036@yzu.edu.cn (N.J.); zhyuhao@hotmail.com (Z.H.); 2 College of Chemistry and Chemical Engineering, Yangzhou University, Yangzhou 225009, China; mx120210061@stu.yzu.edu.cn; 3 Department of the Heart and Great Vessels, Affiliated Hospital of Yangzhou University, Yangzhou 225009, China

## Error in Table and Figure Description

In the original publication [1], there were mistakes in Table 1 as published. Firstly, the molecular structures of screened compound ID 2 (Naldemedine) and compound ID 4 (Norgestrel) were described incorrectly. Secondly, compound ID 8 (Doxycycline) was inconsistent with the structure in Figure S8. The correct name of compound 8 is Simeprevir. The corrected Table 1, along with corresponding corrections in the Table S1 and Figure S8 descriptions, are provided below. The authors state that the scientific conclusions are unaffected. This correction was approved by the Academic Editor. The original publication has also been updated.

## Figures and Tables

**Figure S8:**
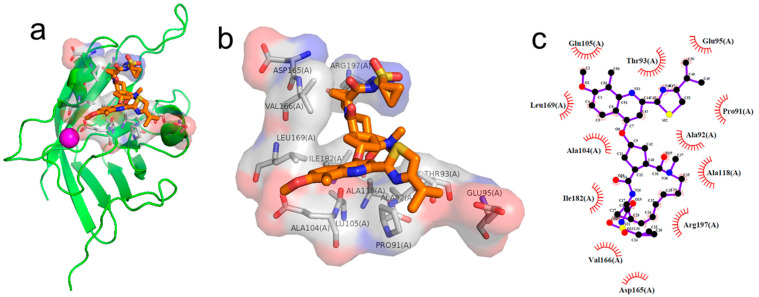
Analysis of the binding pose of Simeprevir with SrtA. (**a**) The structure of the complex. Three-dimensional (**b**) and two-dimensional (**c**) interaction diagrams of the binding pose of Simeprevir with SrtA.

**Table 1:** The molecular docking results indicate that the top 12 drugs have binding energies below −9.0 kcal/mol. The positive and negative controls are depicted in the final two lines. The occupied sites correspond to the binding sites of LPXTG, as shown in Figure 1c.

ID	Compound	Structure	Binding Energy (kcal/mol)	corrScore	Ratio of Occupied Sites
1	Trypan Blue	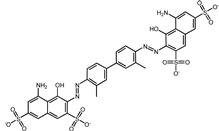	−9.6	0	0
2	Naldemedine	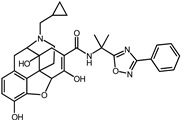	−9.6	0.84	71.4%
3	Lomitapide	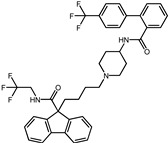	−9.4	0.69	64.3%
4	Norgestrel	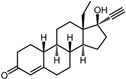	−9.2	0.86	0
5	Triazolam	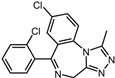	−9.1	0.82	50.0%
6	Flourescein	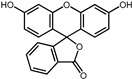	−9.1	0.82	57.1%
7	Midazolam	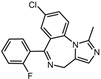	−9.0	0.80	64.3%
8	Simeprevir	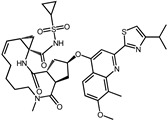	−9.0	0.55	50.0%
9	Alprazolam	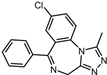	−9.0	0.80	50.0%
10	Telmisartan	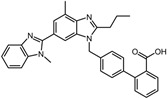	−9.0	0.72	71.4%
11	Nilotinib	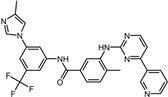	−9.0	0.71	64.3%
12	Azilsartan	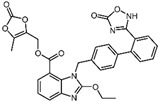	−9.0	0.69	85.7%
Positive control	Rosmarinic acid	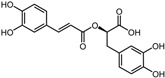	−7.6	0.39	64.3%
Negative control	2,3-Bis(4-methoxyphenyl)propanenitrile	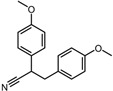	−6.2	0.18	35.7%

**Table S1:** Binding sites of 12 screened drugs and two controls. Residues marked in red are the functional sites of SrtA, as indicated in Figure 1c.

ID	Compound	Binding Sites
1	Trypan Blue	Thr121, Phe122, Ile123, Thr131, Lys134, Asp185, Asp186, Tyr187, Gly192, Val193, Trp194, Lys198
2	Naldemedine	Pro91, Ala92, Leu97, Ala104, Glu105, Glu106, Ala118, Gly119, His120, Val166, Val168, Leu169, Ile182, Cys184, Trp194, Arg197
3	Lomitapide	Pro91, Ala92, Thr93, Leu97, Ala104, Glu105, Glu106, His120, Lys162, Asp165, Val166, Val168, Leu169, Ile182, Arg197
4	Norgestrel	Thr121, Ile123, Asp185, Asp186, Phe122, Trp194, Tyr187
5	Triazolam	Ala92, Ala104, His120, Val168, Leu169, Ile182, Cys184, Arg197
6	Flourescein	Ala104, Ala118, Val161, Lys162, Thr164, Asp165, Val168, Leu169, Ile182, Arg197
7	Midazolam	Ala92, Ala104, His120, Thr164, Asp165, Val168, Leu169, Ile182, Cys184, Arg197
8	Simeprevir	Pro91, Ala92, Thr93, Glu95, Ala104, Glu105, Ala118, Asp165, Val166, Leu169, Ile182, Arg197
9	Alprazolam	Ala92, Ala104, His120, Val168, Leu169, Ile182, Cys184, Arg197
10	Telmisartan	Pro91, Ala92, Thr93, Leu97, Ala104, Glu105, Glu106, Ala118, His120, Lys162, Thr164, Asp165, Val166, Val168, Leu169, Arg197, Ile199
11	Nilotinib	Pro91, Thr93, Ala104, Glu105, Glu106, Asn107, Ala118, Gly119, Lys162, Thr164, Asp165, Val168, Ile182, Arg197, Ile199
12	Azilsartan	Pro91, Ala92, Thr93, Leu97, Ala104, His120, Lys162, Pro163, Thr164, Asp165, Val166, Val168, Val169, Ile182, Cys184, Arg197, Ile199
Positive control	Rosmarinic acid	Leu97, Ala104, Ala118, Gly119, His120, Thr121, Val166, Val168, Leu169, Ile182, Ile183, Cys184, Trp194, Arg197
Negative control	2,3-Bis(4-methoxyphenyl)propanenitrile	Ala92, Leu97, Ala104, Pro163, Val166, Val168, Ieu169
