# Supplementary material for: The Discovery of Novel Agents against Staphylococcus aureus by Targeting Sortase A: A Combination of Virtual Screening and Experimental Validation
Source: Pharmaceuticals (Basel). 2023 Dec 29;17(1):0. doi: 10.3390/ph17010058 (PMC11100315; doi:10.3390/ph17010058)
Supplement: Supplementary file 1 [file pharmaceuticals-17-00058-s001.zip › pharmaceuticals-2939639_Supplementary.pdf]

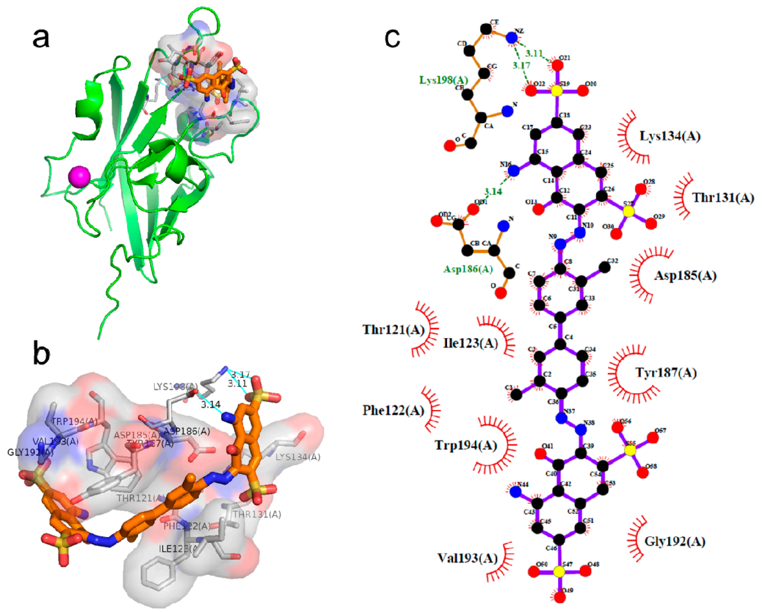

Figure S1. Analysis of the binding pose of Trypan Blue with SrtA. a) The structure of the complex. b-c) 3D (b) and 2D (c) interaction diagrams of the binding pose of Trypan Blue with SrtA.

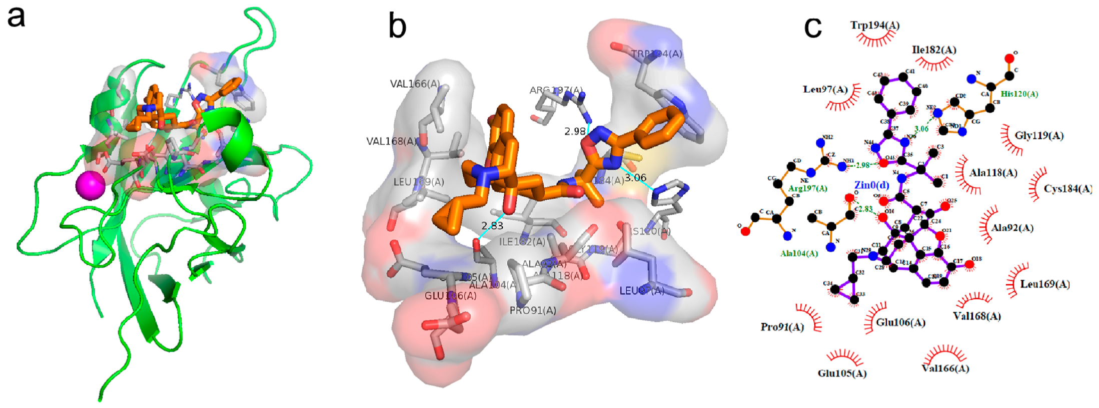

Figure S2. Analysis of the binding pose of Naldemedine with SrtA. a) The structure of the complex. b-c) 3D (b) and 2D (c) interaction diagrams of the binding pose of Naldemedine with SrtA.

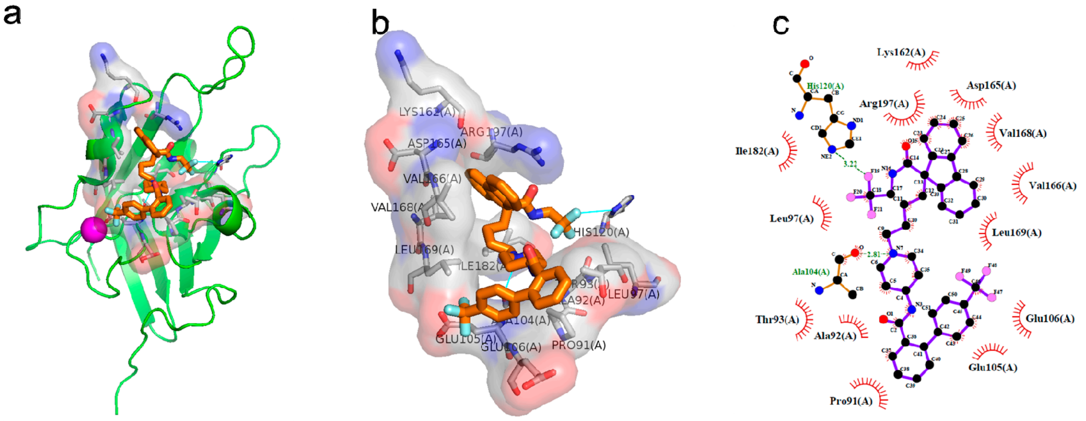

Figure S3. Analysis of the binding pose of Lomitapide with SrtA. a) The structure of the complex. b-c) 3D (b) and 2D (c) interaction diagrams of the binding pose of Lomitapide with SrtA.

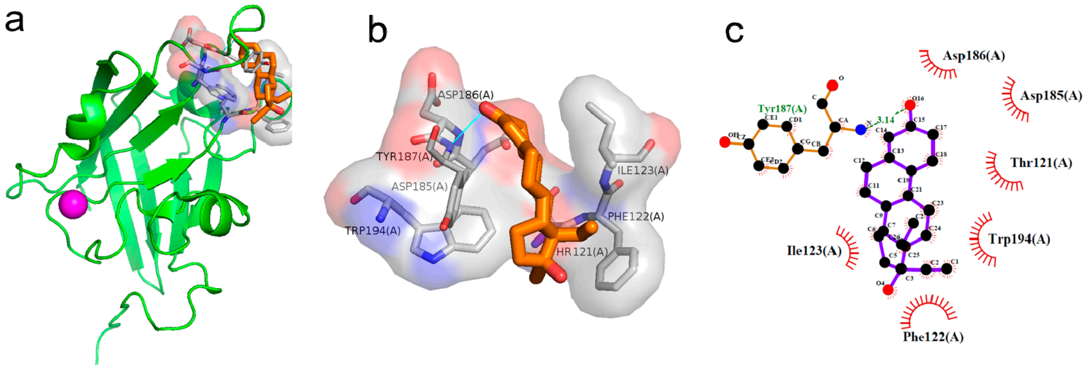

Figure S4. Analysis of the binding pose of Norgestrel with SrtA. a) The structure of the complex. b-c) 3D (b) and 2D (c) interaction diagrams of the binding pose of Norgestrel with SrtA.

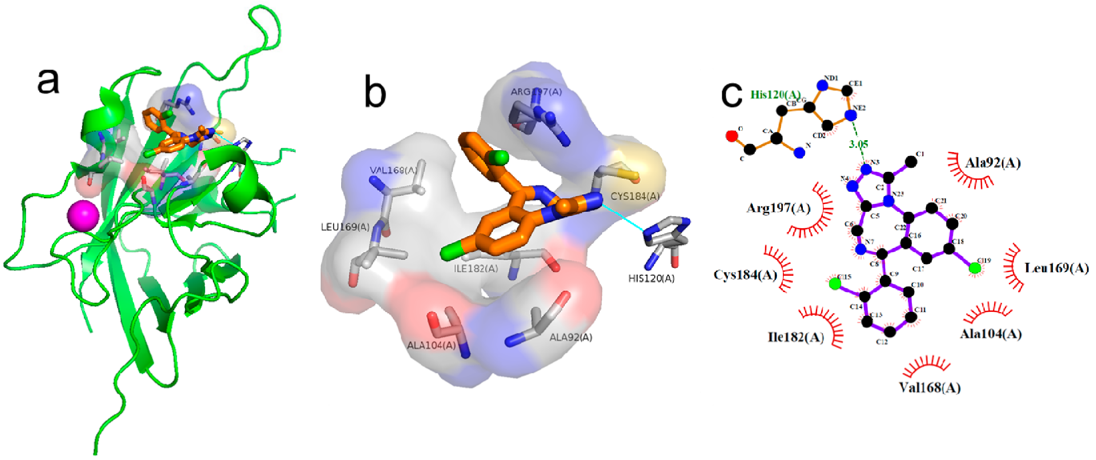

Figure S5. Analysis of the binding pose of Triazolam with SrtA. a) The structure of the complex. b-c) 3D (b) and 2D (c) interaction diagrams of the binding pose of Triazolam with SrtA.

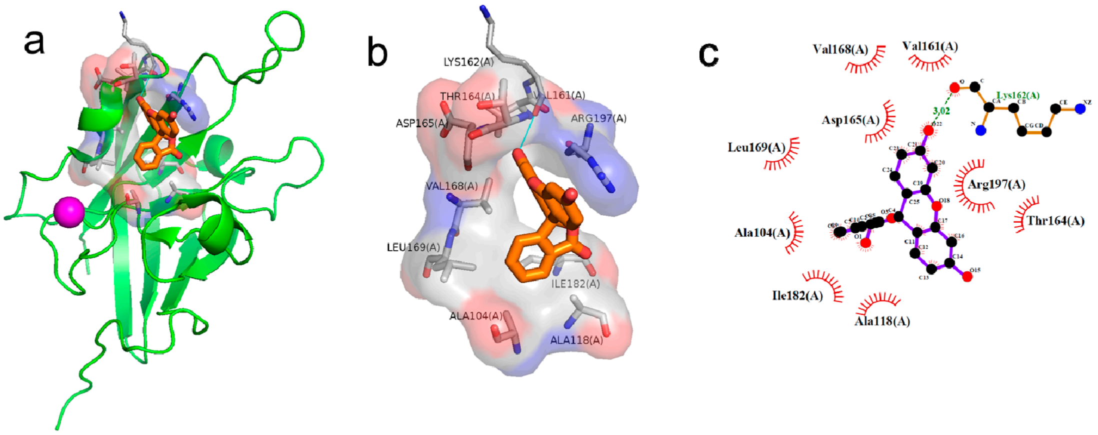

Figure S6. Analysis of the binding pose of Flourescein with SrtA. a) The structure of the complex. b-c) 3D (b) and 2D (c) interaction diagrams of the binding pose of Flourescein with SrtA.

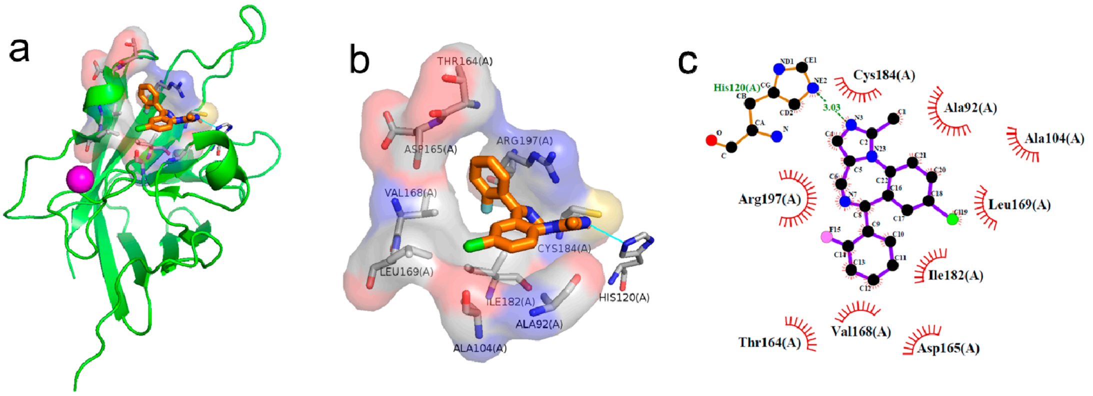

Figure S7. Analysis of the binding pose of Midazolam with SrtA. a) The structure of the complex. b-c) 3D (b) and 2D (c) interaction diagrams of the binding pose of Midazolam with SrtA.

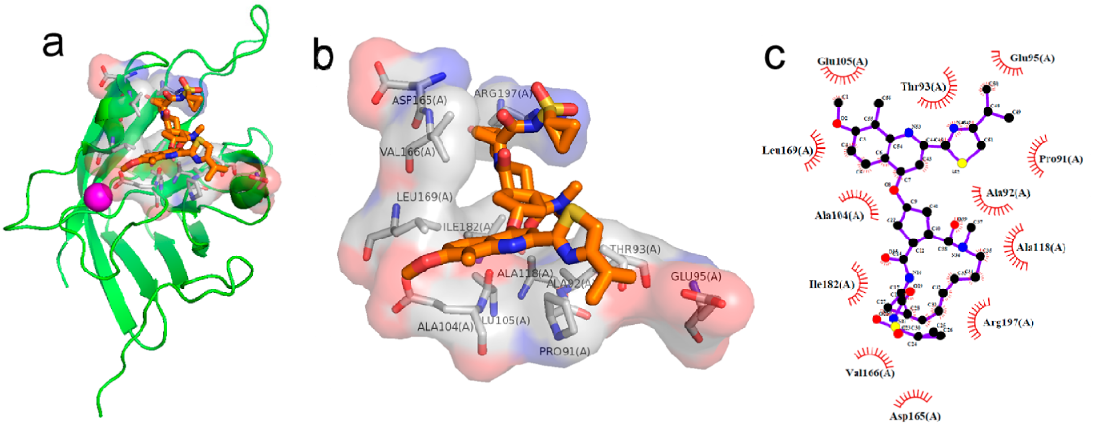

Figure S8. Analysis of the binding pose of Simeprevir with SrtA. (a) The structure of the complex. Three-dimensional (b) and two-dimensional (c) interaction diagrams of the binding pose of Simeprevir with SrtA.

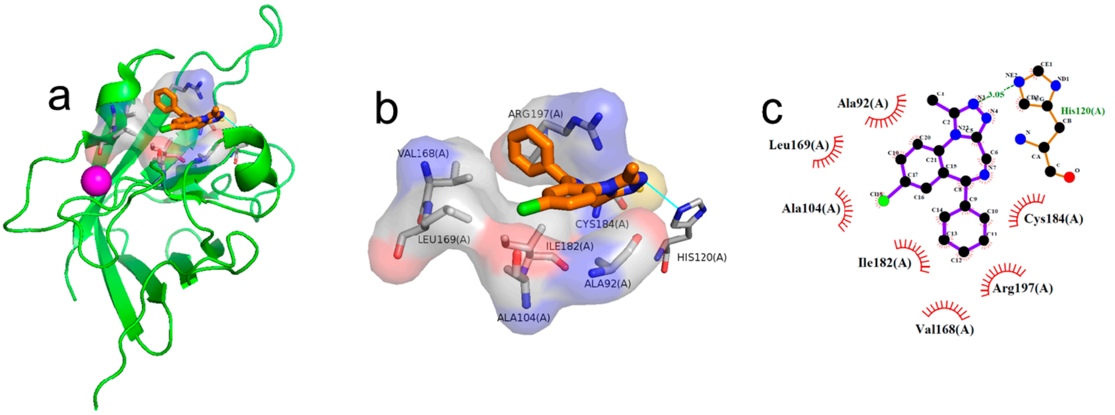

Figure S9. Analysis of the binding pose of Alprazolam with SrtA. a) The structure of the complex. b-c) 3D (b) and 2D (c) interaction diagrams of the binding pose of Alprazolam with SrtA.

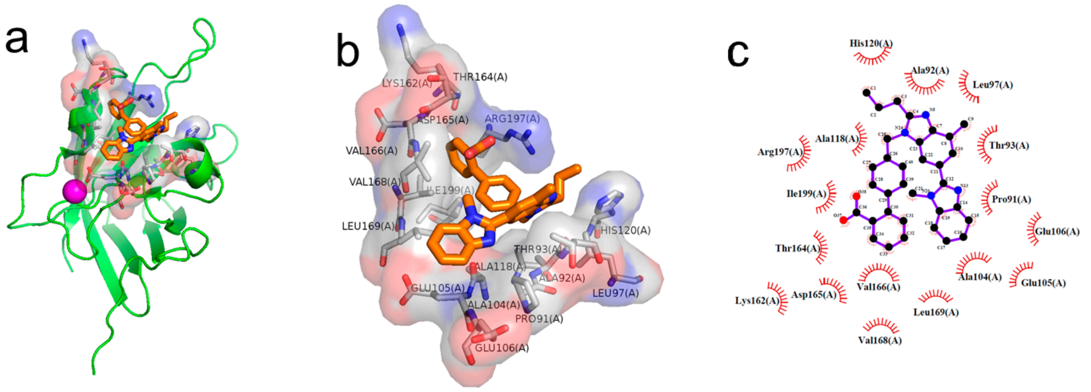

Figure S10. Analysis of the binding pose of Telmisartan with SrtA. a) The structure of the complex. b-c) 3D (b) and 2D (c) interaction diagrams of the binding pose of Telmisartan with SrtA.

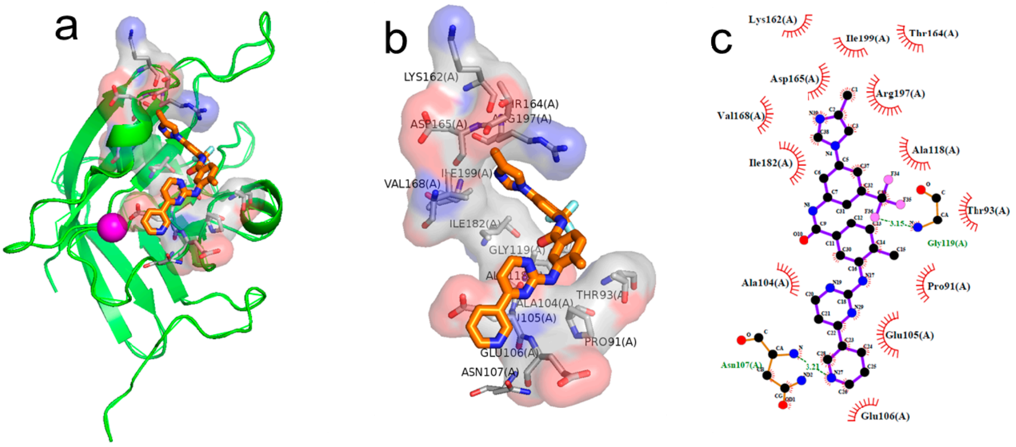

Figure S11. Analysis of the binding pose of Nilotinib with SrtA. a) The structure of the complex. b-c) 3D (b) and 2D (c) interaction diagrams of the binding pose of Nilotinib with SrtA.

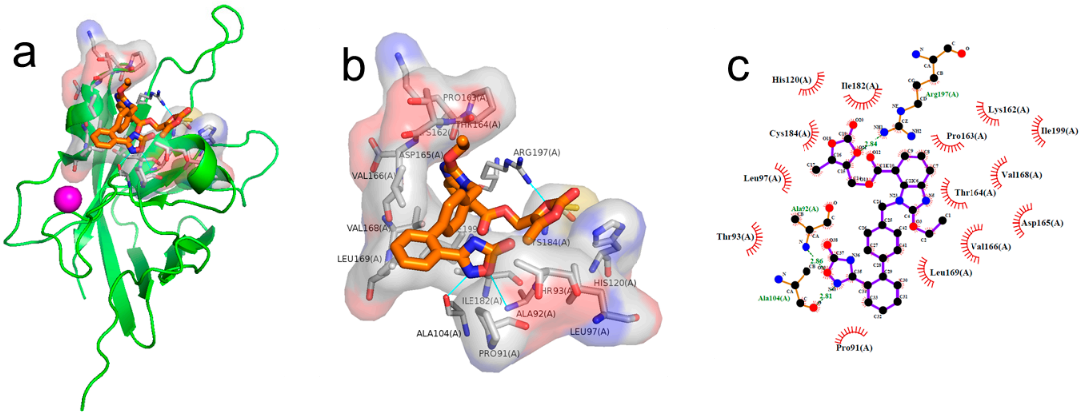

Figure S12. Analysis of the binding pose of Azilsartan with SrtA. a) The structure of the complex. b-c) 3D (b) and 2D (c) interaction diagrams of the binding pose of Azilsartan with SrtA.

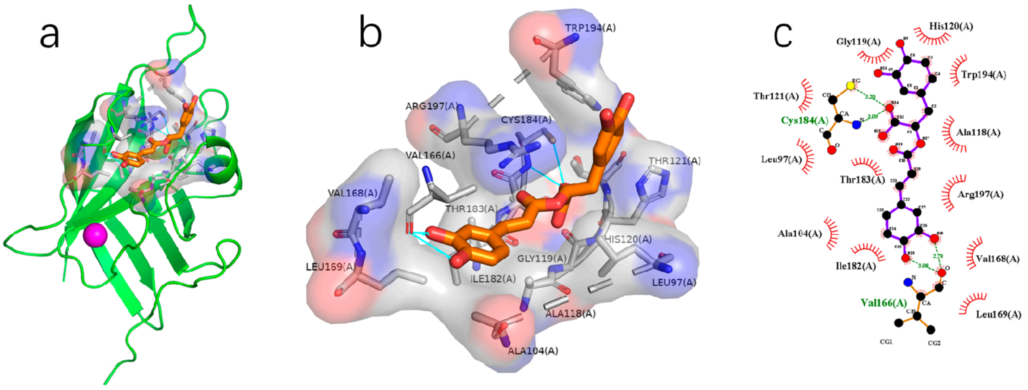

Figure S13. Analysis of the binding pose of positive control with SrtA. a) The structure of the complex. b-c) 3D (b) and 2D (c) interaction diagrams of the binding pose of Rosmarinic acid with SrtA.

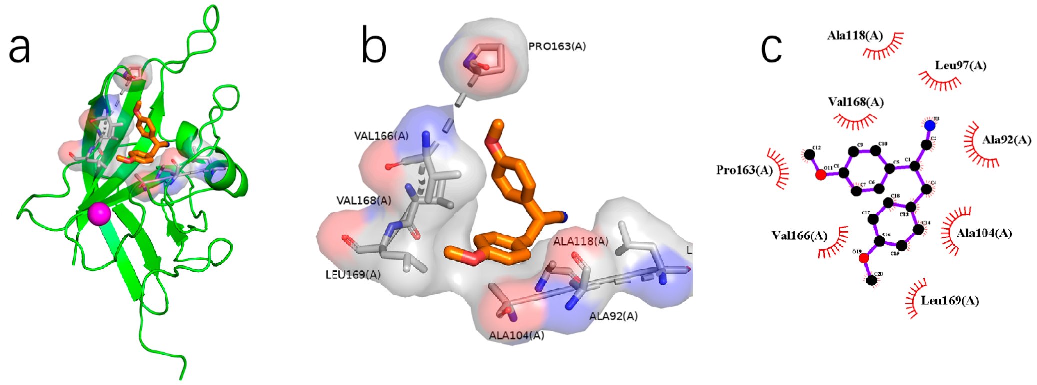

Figure S14. Analysis of the binding pose of negative control with SrtA. a) The structure of the complex. b-c) 3D (b) and 2D (c) interaction diagrams of the binding pose of 2,3-Bis(4-methoxy-phe-nyl)propanenitrile with SrtA.

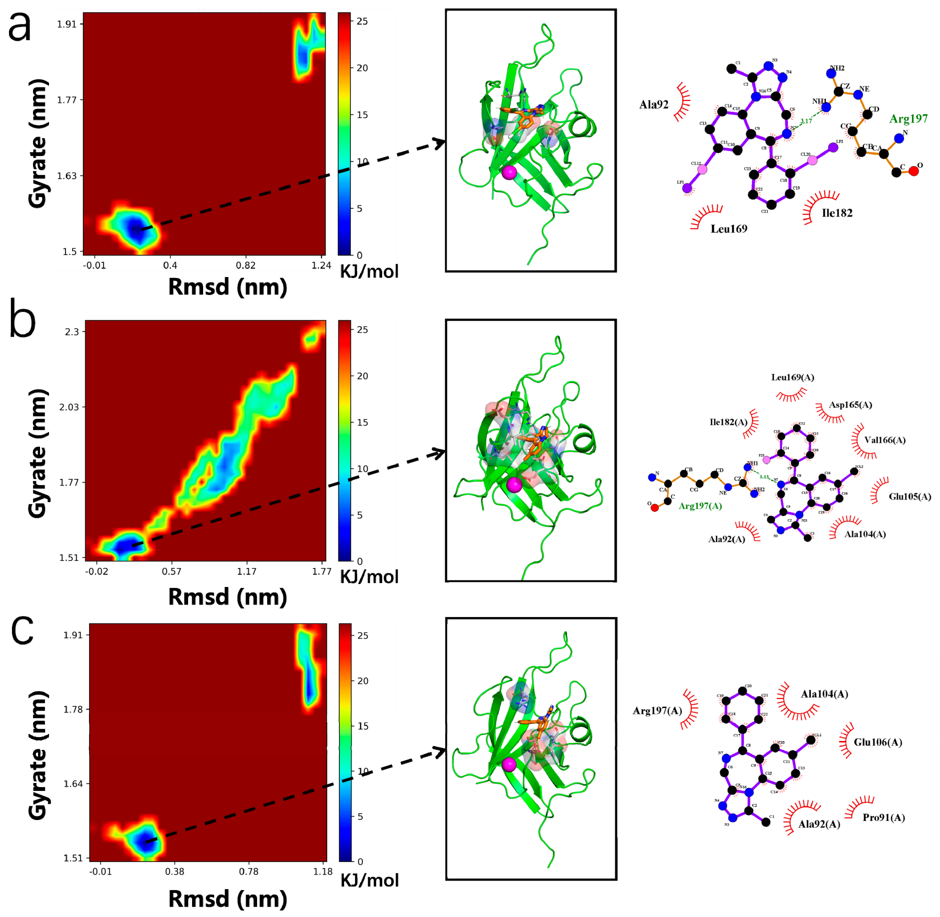

Figure S15. The most stable binding poses of Triazolam (a), Midazolam (b), and Alprazolam (c) with SrtA based on 100 ns MD simulations.

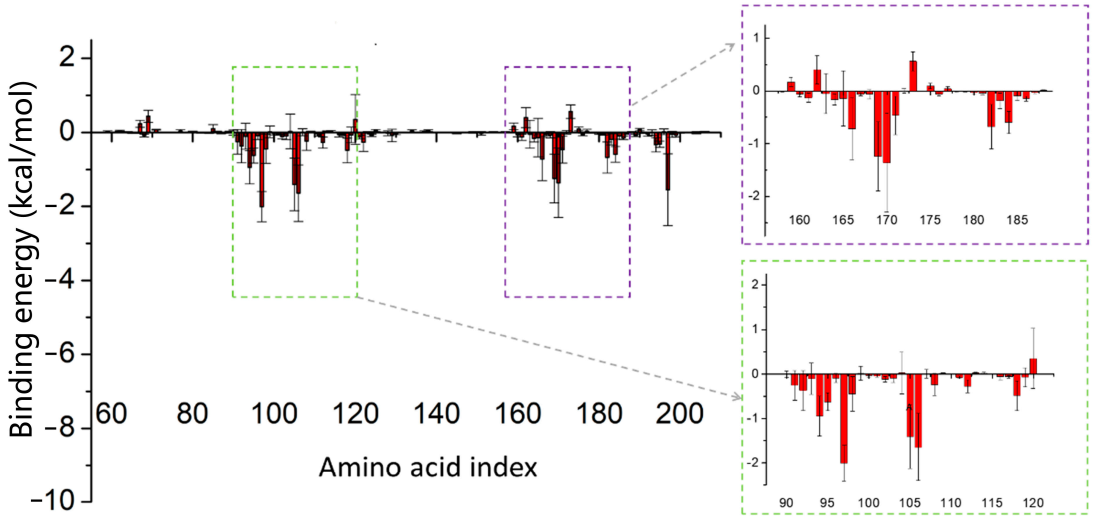

Figure S16. The energy decomposition of all amino acids of SrtA bound with Naldemedine.

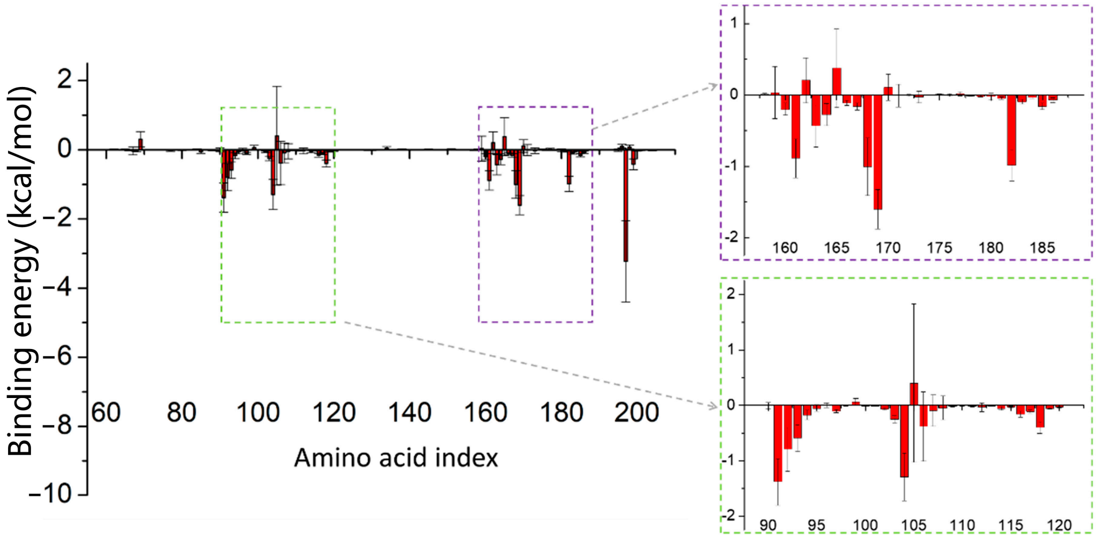

Figure S17. The energy decomposition of all amino acids of SrtA bound with Azilsartan.

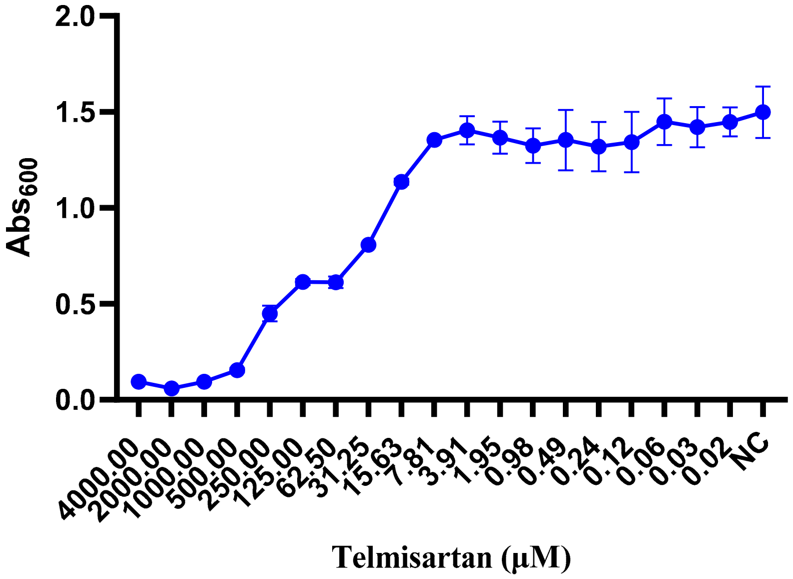

Figure S18. The effect of serial concentrations of Telmisartan on the growth of *S. aureus*.

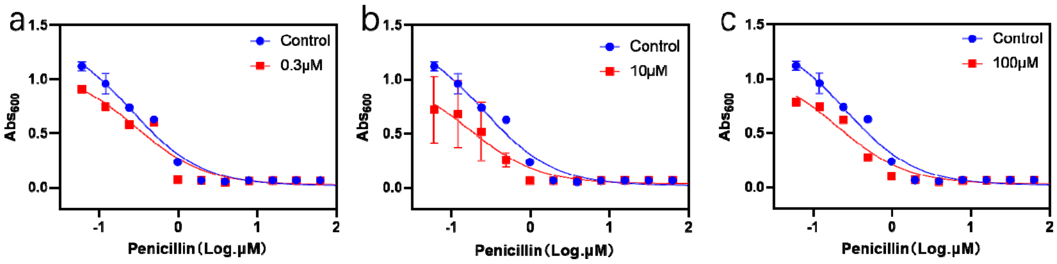

Figure S19. The effect of combination administration on the growth of *S. aureus*. (a) + 0.3 μM Telmisartan, (b) + 10 μM Telmisartan, (c) + 100 μM Telmisartan.

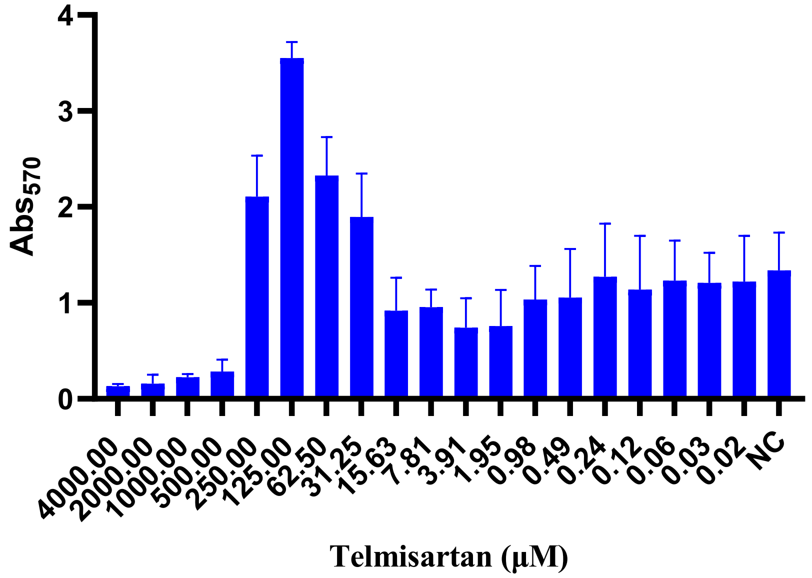

Figure S20. The effect of serial concentrations of Telmisartan on the biofilm formation of *S. aureus*.

Table S1. Binding sites of 12 screened drugs and two controls. Residues marked in red are the functional sites of SrtA, as indicated in Figure 1c.

ID	Compound	Binding sites
1	Trypan Blue	Thr121, Phe122, Ile123, Thr131, Lys134, Asp185, Asp186, Tyr187, Gly192, Val193, Trp194, Lys198
2	Naldemedine	Pro91, Ala92, Leu97, Ala104, Glu105, Glu106, Ala118, Gly119, His120, Val166, Val168, Leu169, Ile182, Cys184, Trp194, Arg197
3	Lomitapide	Pro91, Ala92, Thr93, Leu97, Ala104, Glu105, Glu106, His120, Lys162, Asp165, Val166, Val168, Leu169, Ile182, Arg197
4	Norgestrel	Thr121, Ile123, Asp185, Asp186, Phe122, Trp194, Tyr187
5	Triazolam	Ala92, Ala104, His120, Val168, Leu169, Ile182, Cys184, Arg197
6	Flourescein	Ala104, Ala118, Val161, Lys162, Thr164, Asp165, Val168, Leu169, Ile182, Arg197
7	Midazolam	Ala92, Ala104, His120, Thr164, Asp165, Val168, Leu169, Ile182, Cys184, Arg197
8	Simeprevir	Pro91, Ala92, Thr93, Glu95, Ala104, Glu105, Ala118, Asp165, Val166, Leu169, Ile182, Arg197
9	Alprazolam	Ala92, Ala104, His120, Val168, Leu169, Ile182, Cys184, Arg197
10	Telmisartan	Pro91, Ala 92, Thr93, Leu97, Ala104, Glu105, Glu106, Ala118, His120, Lys162, Thr164, Asp165, Val166, Val168, Leu169, Arg197, Ile199
11	Nilotinib	Pro91, Thr93, Ala104, Glu105, Glu106, Asn107, Ala118, Gly119, Lys162, Thr164, Asp165, Val168, Ile182, Arg197, Ile199
12	Azilsartan	Pro91, Ala92, Thr93, Leu97, Ala104, His120, Lys162, Pro163, Thr164, Asp165, Val166, Val168, Val169, Ile182, Cys184, Arg197, Ile199
Positive control	Rosmarinic acid	Leu97, Ala104, Ala118, Gly119, His120, Thr121, Val166, Val168, Leu169, Ile182, Ile183, Cys184, Trp194, Arg197
Negative control	2,3-Bis(4-methoxyphenyl)propanenitrile	Ala92, Leu97, Ala104, Pro163, Val166, Val168, Ieu169

Table S2. The results of minimum inhibitory concentration (MIC) and fractional inhibitory concentration index (FICI).

	Penicillin				Telmisartan
	Penicillin	+ 0.3 μM Telmisartan	+ 10 μM Telmisartan	+ 100 μM Telmisartan	
MIC (μM)	1.95	0.98(2)	0.98(2)	0.98(2)	1000
FICI		0.5	0.5	0.5	

The fold reduction in the Penicillin MIC in combination with Telmisartan is indicated in brackets. Each value represents the mean of three independent experiments.
